# The first workshop towards the control of cestode zoonoses in Asia and Africa

**DOI:** 10.1186/1756-3305-4-114

**Published:** 2011-06-21

**Authors:** Akira Ito, Munehiro Okamoto, Tiaoying Li, Toni Wandra, Nyoman S Dharmawan, Kadek I Swastika, Paron Dekumyoy, Teera Kusolsuk, Abmed Davvajav, Anu Davaasuren, Temuulen Dorjsuren, Sissay M Mekonnen, Zerihun H Negasi, Tetsuya Yanagida, Yasuhito Sako, Minoru Nakao, Kazuhiro Nakaya, Antti J Lavikainen, Agathe Nkouawa, Tahereh Mohammadzadeh

**Affiliations:** 1 Asahikawa Medical University, Asahikawa, Japan; 2 Institute of Primate Research Institute, Kyoto University, Inuyama, Japan; 3 Sichuan CDC, Chengdu, China; 4 Directorate General Disease Control and Environmental Health, Ministry of Health, Jakarta, Indonesia; 5 Faculty of Veterinary Medicine, Udayana University, Bali, Indonesia; 6 Faculty of Medicine, Udayana University, Bali, Indonesia; 7 Faculty of Tropical Medicine, Mahidol University, Bangkok, Thailand; 8 National Center of Communicable Diseases, Ministry of Health, Ulaanbaatar, Mongolia; 9 Health Science University of Mongolia, Ulaanbaatar, Mongolia; 10 Haramaya University, Dire-Dawa, Ethiopia; 11 Haartman Institute, University of Helsinki, Helsinki, Finland

## Abstract

The first workshop towards the control of cestode zoonoses in Asia and Africa was held in Asahikawa Medical University, Japan on 15 and 16 Feb 2011. This meeting was fully supported by the Asian Science and Technology Strategic Cooperation Promotion Programs sponsored by the Special Coordination Funds for Promoting Science and Technology, the Ministry of Education Japan (MEXT) for 3 years from 2010 to Akira Ito. A total of 24 researchers from 9 countries joined together and discussed the present situation and problems towards the control of cestode zoonoses. As the meeting was simultaneously for the establishment of joint international, either bilateral or multilateral collaboration projects, the main purposes were directed to 1) how to detect taeniasis/cysticercosis infected patients, 2) how to differentiate *Taenia solium *from two other human *Taenia *species, *T. saginata *and *T. asiatica*, 3) how to evaluate *T. asiatica *based on the evidence of hybrid and hybrid-derived adult tapeworms from Thailand and China, 4) how to evaluate *T. solium *and *T. hyaenae *and other *Taenia *species from the wild animals in Ethiopia, and 5) how to detect echinococcosis patients and 6) how to differentiate *Echinococcus *species worldwide. Such important topics are summarized in this meeting report.

## History

The coordinator, Akira Ito at Asahikawa Medical University (AMU) organized a symposium on cysticercosis at the 3^rd ^Seminar on Food-borne Parasitic Zoonoses in Bangkok, 2000 [1]. From 2003, Akira Ito was recommended to establish leadership in science and technology in Asia through his research on cestode zoonoses by a special Japanese Governmental fund for three year project (2003-2005). Akira Ito set up an international symposium every year [2] and seminars for the transfer of technology through joint research projects on cestode zoonoses mainly in Asia and some in Africa. The first seminar was held in AMU from 17 Jan until 2 Feb 2004. We invited 10 guests from Inodonesia (4), Thailand (3), China (3) with international consultants from USA (Peter M Schantz, CDC), Australia (David D Jenkins, the President of Australian Society for Parasitologists) and UK (Philip S Craig, University of Salford, the coordinator for the WHO informal Working Group on Echinococcosis). The second one was held from 14 Sept until 1 Oct 2004. We invited 7 guests from Cameroon (2), Nepal (2), Vietnam (1), Philippines (1) and Australia (1). The third one was held from 17 Jan until 2 Feb 2006. Nine guests were from China (4), Indonesia (2), Philippines (1), Thailand (1) and France (1). The biggest program was to organize the international symposium entitled "Taeniasis/cysticercosis and echinococcosis with focus on Asia and the Pacific" held in the Asahikawa Grand Hotel on 5-8 July 2005. The proceedings of this meeting were published as a supplement of Parasitology International in 2006 [3]. All participants were invited to join together in Asahikawa with full support for travel expenses including air tickets, accommodation and living expenses. Approximately 100 researchers from 30 countries, including Japan, participated. Approximately 80 participants were foreign guests including experts from WHO, FAO and CDC. After this 3 year project, we received another 3 year project for the establishment of the reference center for cestode zoonoses in Asia and Africa from the Japan Society for the Promotion of Science (JSPS) twice continuously (2006-2008, 2009-2011). Akira Ito organized international symposia in Bangkok (2006) at JITMM2006 [4], Okinawa (2007) at the 21^st ^Pacific Science Congress [5-9], Cheju at 17^th ^ICTMM (2008), Bangkok (2009) at JITMM2009, Bangkok (2010) at JITMM2010. In 2011, we also expect to organize it in Bangkok at JITMM2011. These symposia are the major subjects sponsored by JSPS fund from 2006 until 2011.

From 2010, Akira Ito has been recommended to start a special project entitled "Development of molecular and immunological tools for detection of neglected cestode zoonoses" from the Ministry of Education, Japan (MEXT) for three years. This special fund is mainly for development of international collaborative research based on the establishment of the network by the JSPS fund.

## Report

### The first Workshop and seminar for the transfer of technology through joint projects

From 15 February 2011, we organized the first workshop of this new project and also simultaneously started seminars for the transfer of technology through collaboration for 3 weeks. In this report, we would like to introduce what we have been doing through this new project sponsored by the Japanese Government. A total of 23 participants including two PhD students from Cameroon and Iran studying at AMU, joined together from 15 Feb 2011 at AMU. Guests were from China (2), Mongolia (3), Thailand (2), Indonesia (3), Ethiopia (2), Finland (1) and Japan including AMU staff (8).

On 15 and 16 Feb 2011, we had the first workshop with an observer, Takashi Nishigaki, the project coordinator head from Japan Science and Technology Agency (JST), Ministry of Education (MEXT), Japan. After Akira Ito's brief welcoming remarks stressing that "We live together and share the science, technology and philosophy to do joint projects for control of cestode zoonoses", Tiaoying Li and Xingwang Chen from Chengdu, China introduced the present problems of taeniasis/cysticercosis in Sichuan, China with an excellent general overview of taeniasis/cysticercosis [10] and made very clear what they wanted to do on this topic in AMU: 1) seroepidemiology of cysticercosis where we had previously confirmed taeniasis/cysticercosis of *Taenia solium*, taeniasis of both *T. saginata *and *T. asiatica *in the past several years, 2) molecular identification of 51 tapeworms collected in October 2010 through the joint project with the Japanese team (Akira Ito, Munehiro Okamoto, Minoru Nakao, Tetsuya Yanagida, Agathe Nkouawa) and international consultants (Patrick Giraudoux, Francis Raoul from France and Philip S Craig from UK), 3) copro-PCR for 150 stool samples and 4) cysticerci collected from 16 pigs during the new year 2011 to detect hybrid metacestodes of *T. saginata *and *T. asiatica*, and 5) discuss the strategy for transmission ecology of cysticercosis from taeniaisis using GIS [6] which we have been doing as multilateral joint project in China (China, France, UK and Japan) using Japanese funds, with the aim of establishing the current transmission model of *T. solium *in Tibetan communities of Sichuan, China. Hybrid and hybrid-derived tapeworms were already confirmed not only from Thailand [11] but also from China (Yamane et al. in prep.; Nkouawa et al. in prep.).

The second speaker was Toni Wandra. He overviewed the present situation of taeniasis/cysticercosis in Indonesia. Taeniasis of *T. saginata *was rather common but cysticercosis of *T. solium *was very rare and sporadic in Bali, whereas taeniasis/cysticercosis of *T. solium *was still very common in Papua [12-16] and *T. asiatica *was not so rare in Sumatra [12]. Nyoman S Dharmawan presented serological studies of bovine cysticercosis experimentally infected with eggs of *T. saginata*. This is one of the joint projects with Munehiro Okamoto and Akira Ito. Kadek I Swastika presented an important topic from Karang Asem, northeastern part of Bali, where an Indonesian and Japanese joint team worked from 17 until 21 Jan 2011. His main purpose was 1) to investigate serology for people from Karang Asem, and 2) for people randomly taken in Wamena, Papua in July 2010, and 3) undertake molecular identification of *Taenia *samples from both Papua and Bali and also 4) confirm molecular and serological confirmation of one ocular cysticercosis in Bali.

Paron Dekumyoy and Teera Kusolsuk from Thailand focused on taeniasisis/cysticercosis in the border between Thailand and Myanmar [17,18] and reported 4 day field work in Tak province with Munehiro Okamoto and Akira Ito from 5 to 8 Feb 2011, just a few days before this seminar in Asahikawa. His colleagues and the Japanese participants performed microscopy of 150 stool samples and found taeniid eggs from 6 people. So, the main work was to identify the eggs by multiplex PCR and LAMP and do the same work using stool samples.

Abmed Davaajav and Anu Davaasuren from Mongolia summarized taeniasis cases in Mongolia [19]. The main purpose of this project was 1) molecular identification of *Taenia *spp. using multiplex PCR and compare it with copro LAMP, and 2) serodiagnosis for several CE cases. Temuulen Dorjsuren summarized echinococcosis in Mongolia [20,21]. Her main purpose was 1) molecular identification of G1 and G6 genotypes of *Echinococcus granulosus *sensu lato using approximately 50 CE cases and 2) confirm additional one AE case, and 3) serology for both CE and AE. These samples were provided from many collaborators in Ulaanbaatar through Akira Ito's continuous effort to establish a better network for all researchers involved in echinococcosis join together. It was encouraging to hear that Mongolian researchers from different institutions joined together for the "Echinococcosis Working Group in Mongolia" [20,21].

Sissay M Mekonnen from Ethiopia summarized human taeniasis and taxonomy of *Taenia *from wild animals in Ethiopia. His main purpose was to analyze the taeniid worms collected from hyenas, since we were interested in obtaining molecular information on *T. hyaenae *[22]. Zerihun H Negasi presented echinococcosis in domestic and wild animals in Ethiopia. His main purpose was molecular identification of parasite specimens from camels, cattle and goats.

Antti J Lavikainen from Finland gave a brief talk on his interest in molecular phylogeny of Taeniidae including *Taenia *from wild bear in Finland and stressed his main purpose to pursue molecular work on this parasite and several others in Europe and Russia, and join with an Ethiopian project [23]. All Japanese researchers were basically the host scientists and gave brief introductions for their main studies and tools, which they would like to share with the guests. Minoru Nakao spoke on the molecular phylogeny of Taeniidae and molecular evolutional aspects of *Echinococcus *spp. worldwide and explained molecular approaches for parasite samples from all guests [24,25]. Yasuhito Sako gave new tools for serodiagnosis for echinococcosis and cysticercosis. He developed highly specific and sensitive serology for both AE and CE using recombinant antigens including the rapid immunochromatographic kits [26] and for cysticercosis using native and recombinant antigens [27]. This time, he established a novel simple method for purification of diagnostic antigens for cysticercosis (Sako et al., in prep.) and opened it for all guests. Tetsuya Yanagida discussed the haplotype network of *Echinococcus *spp. and *Taenia solium *[28]. Kazuhiro Nakaya summarized the usefulness of experimental animal models for these cestode zoonoses [29]. Munehiro Okamoto talked on the recent topics on the hybrids of between *T. saginata *and *T. asiatica *[11].

**Figure 1:**
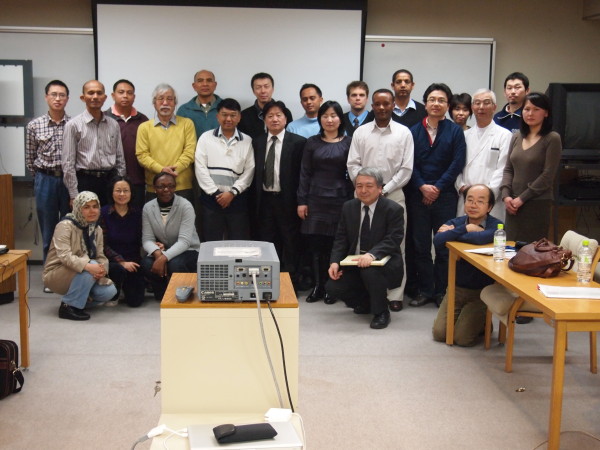
**This is a photo just after the two day workshop at Asahikawa Medical University on 16 February 2011 with consent of all participants**.

Finally, Takashi Nishigaki gave a perspective for the Governmental special fund for future collaboration and stressed that we should continue international collaboration projects and apply for future funding (Figure 1).

So, the levels in science, technology, knowledge and experience in parasitic diseases and evidence-based epidemiological studies were highly variable among the participants from different countries joined together in Asahikawa. However, this was not problematic but encouraged all participants to join together and help one another and improve the curiosity or interest for further studies and collaboration. PhD students from Cameroon and Iran also joined together. Agathe Nkouawa from Cameroon showed how to conduct multiplex PCR and LAMP for taeniasis and cysticercosis of all parasite samples from human or animal origins and copro-PCR and -LAMP for stool examinations, not only in the laboratory but also in the field for the real time identification of the species [30,31]. Tahereh Mohammadzadeh used mitochondrial DNA confirmation for *Echinococcus *spp. and serology for CE.

## Outcome from the seminar from 15 Feb until 4 March 2011

Every Wednesday, we had a 2-3 h seminar to summarize what we had done.

1) China project: Of 51 tapeworms from 35 Tibetan carriers analyzed by multiplex PCR and LAMP, 9 worms from 4 carriers were confirmed as *T. solium*, 40 worms from 30 individuals were identified as *T. saginata*, and the remaining 2 worms from two carriers were confirmed as hybrids of *T. saginata *and *T. asiatica*. Additionally, one of the 35 carriers was identified as dual infection of *T. solium *and *T. saginata*. Serology for cysticercosis in the same Tibetan community revealed that 10.4% (25/240) showed positive response. Further imaging examination is necessary for these sero-positive individuals. Copro-PCR suggested that 35 (23.3%) of 150 stools were positive for *T. solium *or *T. saginata*. Of these 35 positive samples, 25 were verified as taeniasis by collection of tapeworms following treatment with pumpkin seeds combined with areca [32] and/or by microscopy, whereas the remaining 10 cases were newly diagnosed. In other words, copro-PCR could detect more taeniasis carriers in endemic areas.

2) Indonesia project: Although we could not find any *T. solium *carriers in Bali in the past decade, we finally succeeded in detection of several carriers from Karang Asem (Swastika in prep.). All including an immature cysticercus of an ocular cysticercosis case [33] were confirmed to be the Asian genotype of *T. solium *[34] by multiplex PCR and/or LAMP. Molecular identification of *T. solium *from Papua and Bali showed more similarity than we had obtained previously [35]. Further studies are necessary for such work to reach a conclusion on the origin of *T. solium *in Papua. Serology for cysticercosis in Bali and Papua showed critical differences. People in Bali showed relatively weak positive responses from few people, whereas those in Papua showed very strong positive responses from the majority of people [36]. As we know well, cysticercosis is highly endemic in Papua, but it is still sporadic and, therefore, we expect that the control of cysticercosis in Bali may be feasible after the establishment of the strategy for control of cysticercosis by both the central and local Governments. As the field survey has been carried out by the central CDC in Jakarta, Provincial CDC in Bali and Udayana University in Bali, we expect that we can control cysticercosis in Bali. By contrast, it is very difficult to control cysticercosis in Papua within a few decades [9,12,15,16].

3) Thai project: Stool examination by multiplex PCR and LAMP revealed that 6 taeniases were by *T. solium *(n = 5) and one was by *T. saginata*. As the carriers were not treated during our short stay in Feb 2011, we were setting up treatment of the people and additional stool examination in the same village and serology for cysticercosis. [We had a second visit to the same village for treatment of these carriers in May 2011. Unexpectedly, we failed in treatment of three of these carriers, who were basically refugees or immigrants from Myanmar and unable to move out. They moved to urban areas to obtain jobs without treatment. Therefore, these carriers are expected to become high risk persons who may cause secondary cysticercosis in the urban areas in the future.]

4) Mongolian project: All *Taenia *specimens were confirmed as *T. saginata *by multiplex PCR as was confirmed from another project coordinated by Abmed Davaajav in 2006 [17]. *Echinococcus *specimens from CE were confirmed to consist of G1 and G6 as we recognized it in advance in 2006 when Akira Ito invited one junior researcher under Abmed Davaajav [17] and the 6^th ^AE case was confirmed after Bat-Ochir Oyun-Erdene at the Pathology Center, Ministry of Health showed the pathological data in 2010 to Akira Ito [18].

5) Ethiopian project: Molecular approaches for the *Taenia spp. c*ollected from hyenas showed at least three unknown different *Taenia *species. Morphological description is ongoing. We, including Ethiopians, Antti J Lavikainen and Japanese, have a great interest on the origin of human *Taenia *[22], since *T. solium *has a high homology with *Taenia *species found from African wild animals including hyena and others. In addition, parasite cysts from 11 sheep, 19 cattle and 16 camels were analyzed. *E. granulosus *G1 genotype was identified from all host species and *E. canadensis *G6 genotype from camels and cattle. Surprisingly, one cattle cyst represented an unknown *Taenia *species, the adult stage of which was found in hyenas.

## Perspectives

Our research team at AMU has been working on the establishment of immunological and molecular diagnosis of cestode zoonoses, mainly echinococcosis, and taeniasis and cysticercosis. Confirmative studies are necessary for future and further studies for evidence-based control of such cestode zoonoses or any other infectious diseases.

During this period (from 2001 until 2007), four foreign researchers completed their PhD studies in our team: two students from China and one from Brazil were sponsored by the Japanese Government or other international funds (US-NIH RO1), and one sandwich PhD program student, Ronpaku, a researcher from Indonesia, was sponsored by the Japan Society for the Promotion of Science (JSPS). One guest from Cameroon received a Japanese Governmental scholarship to become a PhD student at AMU from 2006 and was awarded a PhD in March 2011. Two guests completed their PhD thesis at Mahidol University and Akira Ito was an overseas examiner for them. In addition, Akira Ito was an overseas PhD examiner at the Jawaharlal Institute of Postgraduate Medical Education and Research, India in 2011 and will become an overseas examiner at Mahidol University for one of the guests who just started his PhD studies on taeniasis/cysticercosis.

Through such joint projects sponsored by MEXT, we continue to encourage colleagues who will actively work for control of such parasitic diseases as the key researchers and establish better networks. Our philosophy may be easily recognized by the authorship of the joint papers in Indonesia, Thailand, China, Mongolia and Cameroon. Basically, we do not become the first author for any of the primary studies from any countries but invite junior colleagues from the counterpart countries especially on the field work in their home countries, except in a few cases.

## Competing interests

The authors declare that they have no competing interests.

## Authors' contributions

AI prepared the outline of the manuscript and delivered it to all participants to improve the contents especially on their own contributions. All participants amended the report and approved the final version of the manuscript with the photo.
